# Optimization of Catechin and Proanthocyanidin Recovery from Grape Seeds Using Microwave-Assisted Extraction

**DOI:** 10.3390/biom10020243

**Published:** 2020-02-05

**Authors:** Jing Chen, W. P. D. Wass Thilakarathna, Tessema Astatkie, H. P. Vasantha Rupasinghe

**Affiliations:** 1 Institute of TCM and Natural Products, Key Laboratory of Combinatorial Biosynthesis and Drug Discovery (Ministry of Education), School of Pharmaceutical Sciences, Wuhan University, 185 East Lake Road, Wuhan 430071, China; chenjingz@whu.edu.cn; 2 Department of Plant, Food, and Environmental Sciences, Faculty of Agriculture, Dalhousie University, Truro, NS B2N 5E3, Canada; ws714884@dal.ca; 3 Faculty of Agriculture, Dalhousie University, Truro, NS B2N 5E3, Canada; astatkie@dal.ca

**Keywords:** flavanols, condensed tannin, *Vitis vinifera*, microwave-extraction, antioxidant, glucosidase, cytotoxicity

## Abstract

Grape seed extract (GSE) is a rich source of condensed flavonoid tannins, also called proanthocyanidins (PACs). The high molecular weight of polymeric PAC limits their biological activity due to poor bioavailability. The present study was undertaken to explore the potential applicability of microwave-assisted extraction (MAE) to convert GSE-PAC into monomeric catechins. A central composite design (CCD) was used to optimize the processing conditions for the MAE. The maximum total yield of monomeric catechins (catechin, epicatechin, and epicatechin gallate) and PAC were 8.2 mg/g dry weight (DW) and 56.4 mg catechin equivalence (CE)/g DW, respectively. The optimized MAE condition was 94% ethanol, 170 °C temperature, and a duration of 55 min. Compared to the results for PACs extracted via conventional extraction (Con) (94% ethanol; shaking at 25 °C for 55 min), MAE yielded 3.9-fold more monomeric catechins and 5.5-fold more PACs. The MAE showed higher antioxidant capacity and α-glucosidase inhibitory activity than that of the conventional extract, suggesting the potential use of the MAE products of grape seeds as a functional food ingredient and nutraceutical.

## 1. Introduction

Catechins, also called flavanols or flavan-3-ols, are a sub-group of flavonoids that are found in some plant-based foods [1]. The commonly found catechins include catechin, epicatechin (EC), epicatechin gallate (ECG), epigallocatechin (EGC), and epigallocatechin gallate (EGCG). Over the past two decades, catechins have attracted interest due to their biological activities, such as antioxidant, antibacterial, and anti-inflammatory properties, among many others [2,3]. Moreover, increasing number of studies have demonstrated that catechins also possess anticarcinogenic activity in many experimental systems and many kinds of organs, including the intestine, lung, liver, pancreas, skin, prostate, breast, and cervix [4,5,6,7,8,9]. The cancer-preventive activity of catechins has been suggested to be due to their antioxidant activity and the modulation of multiple cellular signaling pathways [10,11]. 

Grapes (*Vitis vinifera* L.) are one of the most abundant fruit crops in the world, with an annual production of 69 million metric tons [12]. Grape seeds comprise 5% by mass of grapes and are the major industrial byproducts of grape-processing industries such as grape juice and wine. Grape seeds are a good source of phenolic compounds such as gallic acid, catechin, epicatechin, and proanthocyanidins (PACs, condensed tannins) [12]. Grape seed extracts (GSE) are widely consumed as a dietary supplement based on their potent antioxidant, anticancer, antimicrobial, anti-aging, and anti-inflammatory activities, and are generally recognized as safe by the US Food and Drug Administration (US FDA) [13]. However, PACs with a degree of polymerization over four are not absorbable because of their large molecular size. It has been reported that GSE contains a heterogeneous mixture of PAC monomers (5–30%), oligomers (17–63%), and polymers (11–39%) [14].

In the present study, microwave energy was explored as a tool to enhance the extraction of PAC as well as to increase the generation of monomers through depolymerization of PAC. Microwave-assisted extraction (MAE) is a fast and efficient bioactive extraction method that is based on the direct impact on polar compounds [15,16]. Electromagnetic energy, in the frequency range of 300 MHz to 300 GHz, is transferred to heat following ionic conduction and dipole rotation. MAE has been used to enhance the extraction of active compounds from many plant matrixes, including grape seeds [17,18,19]. MAE heats the matrix internally and externally without a thermal gradient; therefore, biomolecules can be extracted efficiently and protectively [18]. MAE is advantageous over conventional extraction techniques, with improved efficiency, reduced extraction time, rapid and volumetric heating of the absorbing medium, low solvent consumption, higher selectivity of target molecules, and a high potential for automation [19]. However, the effects of MAE conditions such as solvents and temperature on the generation of monomeric catechins have not been reported. Therefore, this study aimed to (1) optimize MAE conditions for the recovery of PACs with maximum monomeric catechins from grape seeds, and (2) assess antioxidant capacity, α-glucosidase inhibitory activity, and cytotoxicity of the MAE products.

## 2. Materials and Methods

### 2.1. Materials and Chemicals

Grape seed powder used in this study was provided by Royal Grapeseed, Milton, NY, USA. The grape seeds were from a mixture of commercial grape varieties of *Vitis vinifera*, *V. labrusca*, and hybrids of native American species with *V. vinifera*. The liquid chromatography standards used for the study were obtained as follows: (−)-epicatechin, (+)-catechin, epigallocatechin (EGC), epicatechin gallate (ECG), epigallocatechin gallate (EGCG), and procyanidin B1 and B2 were from ChromaDex (Santa Ana, CA, USA). High-performance liquid chromatography (HPLC) grade methanol, acetonitrile, and formic acid as well as 2-mercaptoethanol, acetic acid, citric acid, gallic acid, hydrochloric acid, sodium sulfite, and methylcellulose were obtained from Sigma-Aldrich (Mississauga, ON, Canada).

For the cell culture experiments, human fetal hepatic (WRL-68; ATCC^®^ CL-48^TM^) and human hepatocellular carcinoma (HepG2; ATCC^®^ HB-8065^TM^) cells were purchased from American Type Culture Collection (Manassas, VA, USA). Minimum essential medium eagle (MEME), fetal bovine serum (FBS), penicillin-streptomycin, L-glutamine, dimethyl sulfoxide (DMSO), phosphate-buffered saline (PBS), trypan blue stain, and phenazine methosulphate (PMS) were purchased from Sigma-Aldrich (Oakville, ON, Canada). CellTiter 96^®^ AQueous MTS reagent powder was purchased from Promega (Madison, WI, USA). 7-AAD viability staining solution (eBioscience™) was purchased from Thermo-Fisher Scientific (Waltham, MA, USA). The remaining chemicals were obtained from Fisher Scientific (Ottawa, ON, Canada).

### 2.2. Microwave-Assisted Extraction

All MAE experiments were conducted using a MARS model 6^®^ (CEM Corporation, Matthews, NC, USA) microwave-accelerated reaction system. The measurement of microwave absorptivity by various pure components was carried out using a pressure-sealed vessel by heating a given mass of compound at a specific power (800 W) and monitoring the bulk temperature. The optimization of extraction was conducted using a rotor with 75 mL Teflon pressure-sealed vessels. Grape seed powder (0.5 g) was introduced into each tube, together with 5 mL of a solution containing aqueous ethanol (26–94%). Since the maximum temperature that could be used with the present microwave tubes without ethanol leakage through the pressure release vent was 170 °C, a temperature range of 110–170 °C was used in the experiment. Accordingly, the tubes were screw-capped, shaken evenly, and placed in the rotor to allow temperature measurement by the combination of fiber optic and infrared sensor, and heated at a given temperature (110–170 °C) for 5–55 min. No stirring was applied during the heating. The temperature program consisted of a fast heating step using a fixed maximum power (800 W), followed by a plateau step during which power varied to maintain the temperature at the target value. At the end of the reaction, samples were allowed to cool down to room temperature (30 min). The solid residue was isolated by filtration. Each experiment was conducted in duplicate. The filtrate was stored at 4 °C until further analysis.

### 2.3. Experimental Design

Response surface methodology (RSM) was used to determine the optimal processing conditions for the MAE extraction of PAC from grape seeds powder. A central composite design (CCD) using 20 runs at low-axial, low, center, high, and high-axial levels of the three factors, namely temperature (110 °C, 120 °C, 140 °C, 160 °C, and 170 °C), ethanol concentration (26%, 40%, 60%, 80%, and 94%), and time (5 min, 15 min, 30 min, 45 min, and 55 min) was generated and analyzed using Minitab 18 software to determine the optimum settings of the factors that maximize two response variables (total monomeric catechins and total PAC content).

### 2.4. Extractions Using the Conventional Method

For comparison, PACs were also extracted from grape seed powder using the conventional method (extraction conditions of 94% (*v/v*) ethanol, 55 min, and 25 °C) without applying microwaves. Grape seed powder (1 g) was introduced into the tube together with 10 mL of 94% ethanol. The tube was capped and shaken at 70× *g* at room temperature for 55 min. The experiment was conducted in triplicate.

### 2.5. Quantification of PAC Content of Extracts 

The total PAC content of the extracts was quantified using the methylcellulose precipitable (MCP) tannin assay described by Dambergs et al. [19]. Methylcellulose solution (0.04% *w/v*; 1500 centipoises viscosity at 2%) was prepared. The samples and catechin standards were prepared with a 50% aqueous ethanol solution. In a 1.5 mL centrifuge tube, 300 μL of methylcellulose solution was mixed thoroughly with a 100 μL sample and 5 min was allowed for completion of the polymerization reaction. Following the addition of 200 μL of saturated ammonium sulfate solution, the sample was made up to 1 mL final volume with deionized water and the solution was vortexed. Centrifugation was performed at 10,000× *g* for 5 min. The control samples were prepared with the same volume as per the treatment sample without methylcellulose solution. After transferring 200 µL of solution from each tube into a UV-transparent 96 well plate, the absorbance at 280 nm was measured. Results were expressed in mg catechin equivalence per gram dry weight of the sample (mg CE/g DW). 

### 2.6. UPLC-ESI-MS Analysis of Catechins in Extracts

Each extract was filtered through a 0.22 μm nylon filter and placed into amber vials. The analyses were performed using a Waters H-class UPLC separations module (Waters, Milford, MA, USA), coupled with a Quattro Micro API MS/MS system and controlled with a Masslynx V4.1 data analysis system (Micromass, Cary, NC, USA). The column used was an Aquity BEH C18 (2.1 × 100 mm, 1.7 μm) (Waters, Milford, MA, USA). For the separation of the catechin, epicatechin, epicatechin gallate (EGC), and epigallocatechin gallate (EGCG), a gradient elution was carried out with 0.1% formic acid in water (solvent A) and 0.1% formic acid in acetonitrile (solvent B) at a flow rate of 0.3 mL/min. A linear gradient profile was used, with the following proportions of solvent A applied at time t (min) (t, A%): (0, 94%), (2, 83.5%), (2.61, 83%), (2.17, 82.5%), (3.63, 82.5%), (4.08, 81.5%), (4.76, 80%), (6.75, 20%), (8.75, 94%), (12, 94%). Electrospray ionization in negative ion mode (ESI-) was used with the following conditions: capillary voltage 3000 V, cone voltage 40 V, and nebulizer gas (N_2_) temperature 375 °C at a flow rate of 0.3 mL/min. Single-ion monitoring (SIM) mode using specific parent ions was employed for quantification in comparison with standards: *m/z* 289 for catechin and epicatechin, *m/z* 442 for ECG, *m/z* 472 for EGCG. The quantification of each analysis was performed using calibration curves created using the external standards. The limit of detection of the analytes was between 0.01 and 0.1 mg/L.

### 2.7. Total Antioxidant Capacity of the Extracts by FRAP Assay

The Ferric Reducing Antioxidant Power (FRAP) assay was performed as previously described by Benzie and Strain [20]. Briefly, the reagents included 300 mM acetate buffer pH 3.6, 40 mM hydrochloric acid, 10 mM 2,4,6-tripyridyl-s-triazine (TPTZ) solution, and 20 mM ferric chloride solution. The working FRAP reagent was prepared fresh on the day of analysis by mixing acetate buffer, TPTZ solution, and ferric chloride solutions in the ratio 10:1:1 and incubating at 37 °C. Absorbance was recorded at 593 nm. The calculated difference in absorbance is proportional to the ferric-reducing antioxidant power of the antioxidants present in the extracts. For quantification, a calibration curve of Trolox (0.1 mM to 1 mM) was used. The final results were expressed as mmol Trolox equivalents per L of sample. The analysis was performed in triplicate.

### 2.8. Inhibitory Effect of Extracts on α-Glucosidase In Vitro

The α-glucosidase-inhibitory assay was performed via the chromogenic method described by Watanabe et al. [21]. Briefly, α-glucosidase (1 U/mL, Sigma) was dissolved in 100 mM phosphate buffer (pH 6.8) containing 0.2% bovine serum albumin used as an enzyme solution. Next, 5 mM 4-nitrophenyl-a-d-glucopyranoside (PNPG) in the same buffer (pH 6.8) was used as a substrate solution. Measures of 20 μL of enzyme solution and 120 μL of strand drug/extract were mixed in a microtiter plate and incubated at 37 °C. After incubation for 15 min, the substrate solution (20 μL) was added and incubated at 37 °C for 15 min. Eighty microliters of 0.2 M sodium carbonate solution was added to stop the reaction. Absorbance was recorded at 405 nm to quantify the amount of PNP released.

### 2.9. Cell Culture

WRL-68 and HepG2 cells were cultured in MEME supplemented with 10% FBS, 4 mM L-glutamine, 100 U/mL penicillin, and 100 μg/mL streptomycin, as described in Thilakarathna and Rupasinghe [22]. Cell cultures were maintained at 37 °C and 5% CO_2_ in a humidified incubator and subcultured before reaching confluence.

### 2.10. Cytotoxicity of PAC in WRL-68 and HepG2 Cells

Cytotoxicity of the PACs extracted by microwave method (MW-PAC) and conventional extraction method (Con-PACs) was evaluated in WRL-68 normal cells and HepG2 hepatocarcinoma cells to compare the cytotoxicity of PAC extracts. Cell viability (%) of the two cell lines was tested over a broad concentration range of MW-PACs and Con-PACs using the MTS cell viability assay and 7-AAD-stained flow cytometry analysis [22]. EGCG was used as a reference phenolic compound in both cell viability experiments. 

#### 2.10.1. MTS Cell Viability/Metabolic Activity Assay

Cells were seeded in a 96 well plate at a density of 6000 cells/well and incubated overnight at 37 °C and 5% CO_2_ in a humidified incubator. Cells were treated with 10–1000 µg/mL concentrations of MW-PAC, Con-PAC, and EGCG for 24 h. Treated cells were exposed to MTS/PMS solution (MTS, 333 μg/mL; PMS, 25 μM of final concentration) and incubated for 3 h at 37 °C. After incubation, absorbance was measured at 490 nm using the Infinite^®^ M200 PRO multimode microplate reader (Tecan Trading AG, Mannedorf, Switzerland).

#### 2.10.2. 7-AAD-Stained Flow Cytometry for Cell Viability/Cell Death Evaluation

WRL-68 and HepG2 cells were seeded in a six-well plate at a density of 2 × 10^5^ cells/well and incubated at 37 °C and 5% CO_2_ in a humidified incubator overnight. Cells were treated with 10 to 1000 µg/mL concentrations of MW-PAC, Con-PAC, or EGCG for 24 h. The culture media was collected into separate tubes from each well to include potential dead cells in the flow cytometric analysis. Each well was then washed with 1 mL of PBS, and cells were harvested with TrypLE express (1 mL/well, incubated for 5 min at 37 °C). PBS (from well washing), TrypLE express, and harvested cells were pooled together with the culture media collected earlier. Samples were centrifuged at 500× *g* for 5 min, and the supernatant was discarded. The cell pellet was washed with cold PBS (500× *g* for 5 min) and re-suspended in 1 mL of PBS. Cells were stained with 5 µL of 7-AAD stain in the dark for 5 min. Samples were analyzed by flow cytometer under the FL-3 filter (BD Accuri^TM^ C6 Plus Flow Cytometer, BD Biosciences, San Jose, CA, USA) and data were processed by Kaluza Analysis (version 2.1) FACS analysis software (Beckman Coulter Life Sciences, Indianapolis, IN, USA).

### 2.11. Statistical Analysis

Complete analyses of total monomeric catechins and total PAC content measured from the 20 runs (including six center points to allow the estimation of error variance) of the central composite design (CCD) conducted in random order were done. The analyses included verification that the model did not have a significant lack of fit (*p* > 0.05), which indicated the adequacy of the model to accurately predict the variation, and the normal distribution and constant variance assumptions on the error terms were valid. Independence assumption was validated through the randomization of the run order. This was followed by testing the significance of each term and constructing contour plots for each response variable to determine the best setting of the factors, and finally determining the “sweet spot” that optimized the two response variables using overlaid contour plots and the response optimizer. These statistical analyses were completed using methods described previously [23,24]. Linear regression analyses were conducted to determine the total PAC content and antioxidant capacity. Prism 6 software (GraphPad Software, La Jolla, CA, USA) was used to calculate IC_50_ values for the inhibitory effect on α-glucosidase and cell viability.

## 3. Results

### 3.1. Response Surface Modeling

#### 3.1.1. Model Fitting of Parameters Based on Total Monomeric Catechins and Total PAC Content

A total of 20 runs were performed to optimize the three factors (temperature, ethanol concentration, and microwave extraction time) in the current CCD. Preliminary single-factor tests were conducted to determine the effective ranges for each of the three factors to be optimized. The maximum temperature that could be used with the present microwave tubes without any ethanol leakage through the pressure release vent was 170 °C. Thus, a temperature range of 110–170 °C was selected for the optimization. Reaction time was distributed around 30 min to ensure complete reactions. Ethanol concentrations ranged between 20% and 100% in order to select the optimum concentration.

The actual values of the three factors (temperature, ethanol concentration, and microwave extraction time) and experimental results for the yield of total monomeric catechins (catechin, epicatechin, and EGC) and total PACs are shown in Table 1. The obtained data were used for the prediction of an optimum set of extraction settings from grape seed powder extract with high monomeric catechins and PAC. These experimental results were fitted to a second-order response surface model, and the analysis of variance (ANOVA) *p*-values that show the significance of the components of the model are presented in Table 2. According to the ANOVA results, the model described the variability in total monomeric catechins and total PACs very well, with adjusted R^2^ values of 85.2% and 94.2%, respectively; and without significant lack of fit (*p* > 0.05). 

#### 3.1.2. Optimization of the MAE Operating Conditions

**Figure 1:**
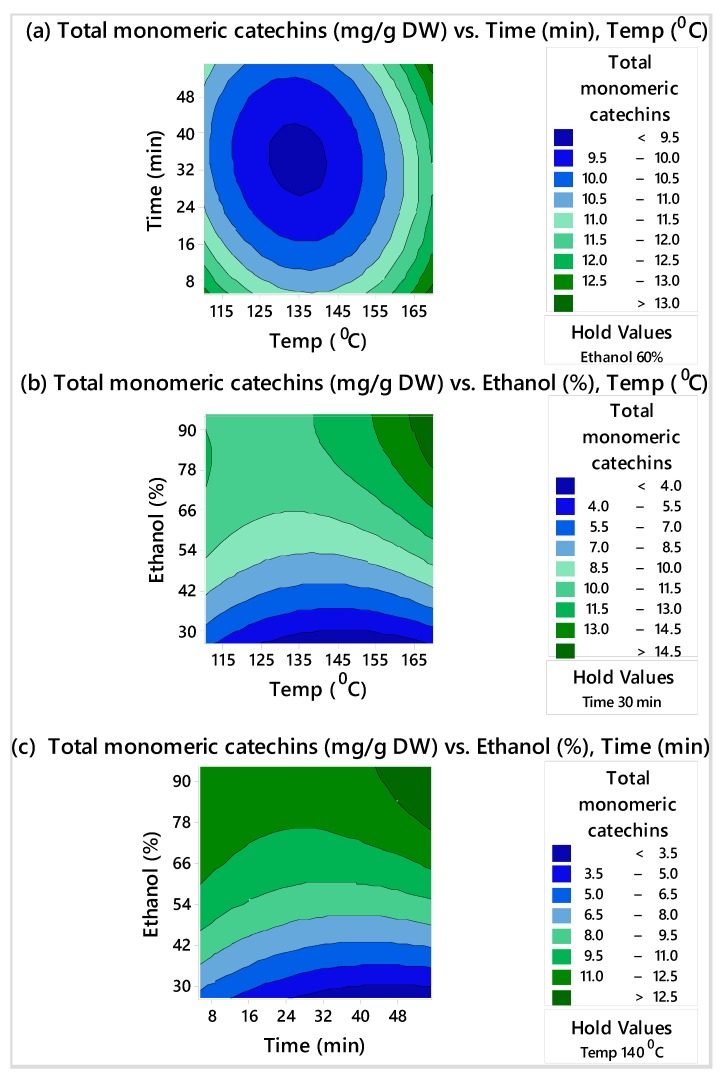
Contour plots of total monomeric catechins (mg/g DW) vs. (**a**) time (min) and temperature (°C) with ethanol held at 60%, (**b**) ethanol (%) and temperature (°C) with time held at 30 min, and (**c**) ethanol (%) and time (min) with temperature held at 140 °C.

**Figure 2:**
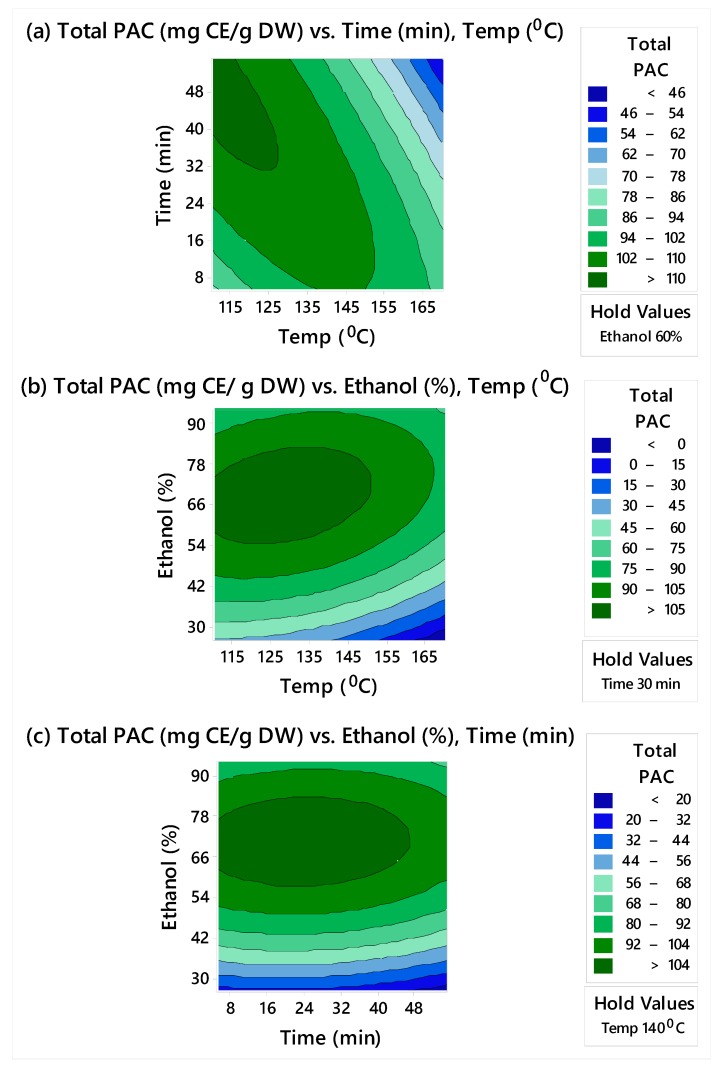
Contour plots of total PACs (mg CE/g DW) vs. (**a**) time (min) and temperature (C) with ethanol held at 60%, (**b**) ethanol (%) and temperature (°C) with time held at 30 min, and (**c**) ethanol (%) and time (min) with temperature held at 140 °C.

Contour plots for yield of total monomeric catechins and total PACs are shown in Figure 1 and Figure 2, respectively. These plots represent how total monomeric catechins and total PACs changed as microwave time and temperature changed while keeping the ethanol concentration constant at 60% (Figure 1a and Figure 2a), as ethanol concentration and temperature changed while the microwave time was kept constant at 30 min (Figure 1b and Figure 2b), and as ethanol concentration and time changed while keeping the temperature constant at 140 °C (Figure 1c and Figure 2c). The strongest effect was attributed to the ethanol concentration, in agreement with the poor solubility of PACs in water. As a consequence, reducing the proportion of ethanol below 60% (*v/v*) decreased the amount of PACs extracted. Moreover, higher ethanol concentrations showed a significant influence on the yield of monomeric catechins from grape seed powder. Microwave temperature and duration showed some influence on the yield of monomeric catechins and PACs. Higher yields of monomeric catechins and PACs were observed when higher temperatures and microwave time were used.

**Figure 3:**
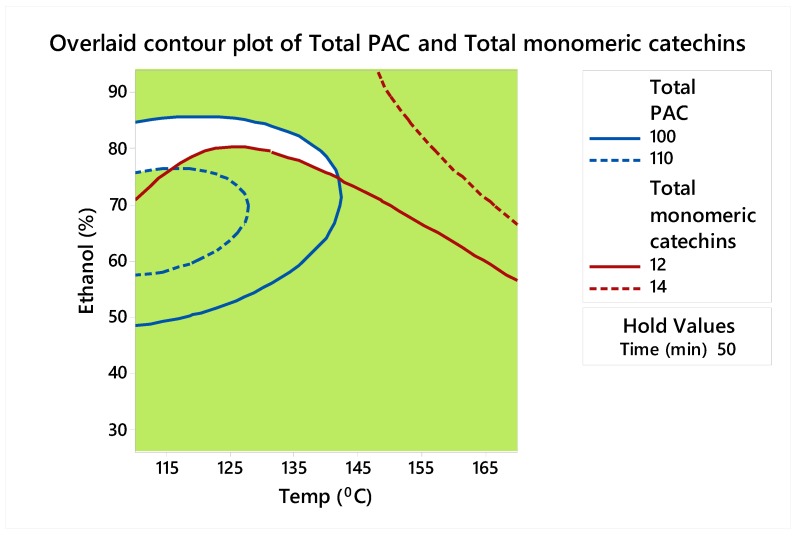
Overlaid contour plot showing the sweet spot (white area) for total PACs (mg CE/g DW) between 100 and 110 mg CE/g DW and total monomeric catechins (mg/g DW) between 12 and 14 mg/g DW.

The contour plots shown in Figure 1 and Figure 2 reflect our focus on determining the optimum settings of the factors to maximize total PACs and total monomeric catechins individually. From these figures, high values of total PACs ranged from 100 to 110 mg CE/g DW, and high values of total monomeric catechins ranged from 12 to 14 mg/g DW. Therefore, an overlaid contour plot that showed the optimum extraction conditions to maximize total monomeric catechins and total PACs jointly within these ranges was produced and is shown in Figure 3. As shown in Table 3, since the optimum times that maximized total PACs and total monomeric catechins were different (41 min and 55 min, respectively), for the overlaid plot (Figure 3), time was held at 50 min (half-way between 41 min and 55 min). Depending on what the objectives of the process are, various conditions may be preferable. 

Response optimizer analysis results that show the optimum settings of temperature, ethanol concentration, and time to maximize total monomeric catechins and total PACs are shown in Table 3. Accordingly, the optimum settings for maximizing total monomeric catechins to 18.3 mg/g DW were 170 °C temperature, 94% ethanol concentration, and 55 min. However, in this setting, the predicted total PACs was 43.7 mg CE/g DW. The optimum setting for maximizing total PAC to 113.6 mg CE/g DW was 120 °C temperature, 68% ethanol concentration, and 41 min. However, in this setting, the predicted total monomeric catechins reduced to 10.7 mg/g DW. 

### 3.2. Comparison with Conventional Extraction

#### 3.2.1. Quantitative Measurement

**Figure 4:**
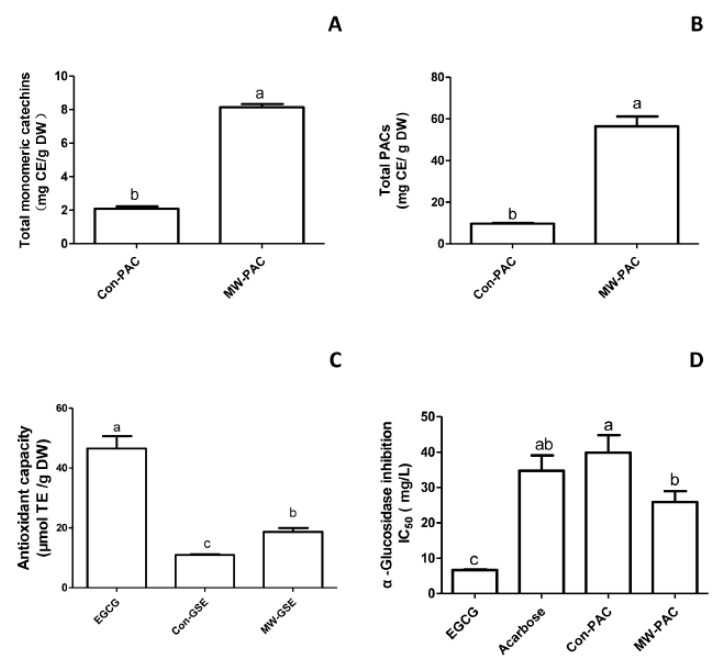
The total monomeric catechins (**A**), total PACs (**B**), total antioxidant capacity (FRAP method) (**C**), and inhibition of α-glucosidase (**D**) of Con-PAC and MW-PAC. The total PAC content is expressed in mg catechin equivalence (CE)/g DW of the sample. Epigallocatechin gallate (EGCG), and acarbose were used for the comparison purpose. Means sharing the same letter are not significantly different at the 5% level.

To determine whether microwaves played an important role in the reactivity and selectivity of the process, we compared the yields and characteristics of the extracts obtained using microwave irradiation (MW) and conventional extraction (Con) under similar conditions: 94% EtOH; shaking at 100× *g*, 55 min; 170 °C for MW and 25 °C for Con. The yields of monomeric catechins and total PAC from grape seed by MW (MW-PAC) and from grape seed powder by Con (Con-PAC) are presented in Figure 4A,B. Total monomeric catechins of Con-PAC consisted of catechin, epicatechin, and EGC at 0.95, 1.04, and 0.11 mg/g DW, respectively. Under conventional conditions (94% EtOH; 25 °C; 55 min), the values for total monomeric catechins and PAC were 2.10 ± 0.13 mg/g DW and 9.70 ± 0.39 mg CE/g DW, respectively. Total monomeric catechins of MW-PAC consisted of catechin, epicatechin, and EGC at 4.0, 3.32, and 0.83 mg/g DW, respectively. Under microwave extraction conditions (94% ethanol; 170 °C; 55 min), the values for total monomeric catechins and PAC were 8.15 ± 0.20 mg/g DW and 56.37 ± 8.37 mg CE/g DW, respectively.

#### 3.2.2. Total Antioxidant Capacity 

Accumulating evidence demonstrates that flavanols, including catechins and PACs, which have high antioxidant capacity, help to decrease the risk of developing chronic diseases including cancer, cardiovascular disease, and diabetes [12,25,26]. Therefore, plant-based therapeutics present potential alternative therapies that should be explored due to their reported safety and health benefits. A FRAP assay was performed to compare the total antioxidant capacity of the extracts by microwave irradiation (MW-PAC) and conventional extraction (Con-PAC). EGCG was used as a reference compound (Figure 4C). MW-PAC (186.9 ± 12.3 µmol TE/L) had greater FRAP values than Con-PAC (109.6 ± 2.4 µmol TE/L). Results indicated that the overall antioxidant capacity was higher in MW-PAC as compared to the corresponding Con-PAC. Pearson’s correlation coefficient was calculated to evaluate the relationship between total PACs and antioxidant capacity. The total PACs of Con-PAC and MW-PAC all showed significant linear correlation (r = 0.9984; *p* < 0.0001, r = 0.9984; *p* < 0.0001, respectively) with FRAP values.

#### 3.2.3. α-Glucosidase-Inhibitory Activity

EGCG, acarbose (the drug), Con-PAC, and MW-PAC were tested for their ability to inhibit α-glucosidase activity (Figure 4D). Acarbose showed an IC_50_ value of 347.4 ± 43.5 mg/L. EGCG exhibited the highest inhibitory activity with the lowest IC_50_ value of 66.9 ± 1.6 mg/L. Compare to the acarbose, Con-PAC and MW-PAC showed comparable inhibitory activities against α-glucosidase (IC_50_: 399.1 ± 48.9 mg/L and 259.0 ± 30.8 mg/L, respectively). 

#### 3.2.4. Cytotoxicity of Con-PAC and MW-PAC in WRL-68 and HepG2 Cells

**Figure 5:**
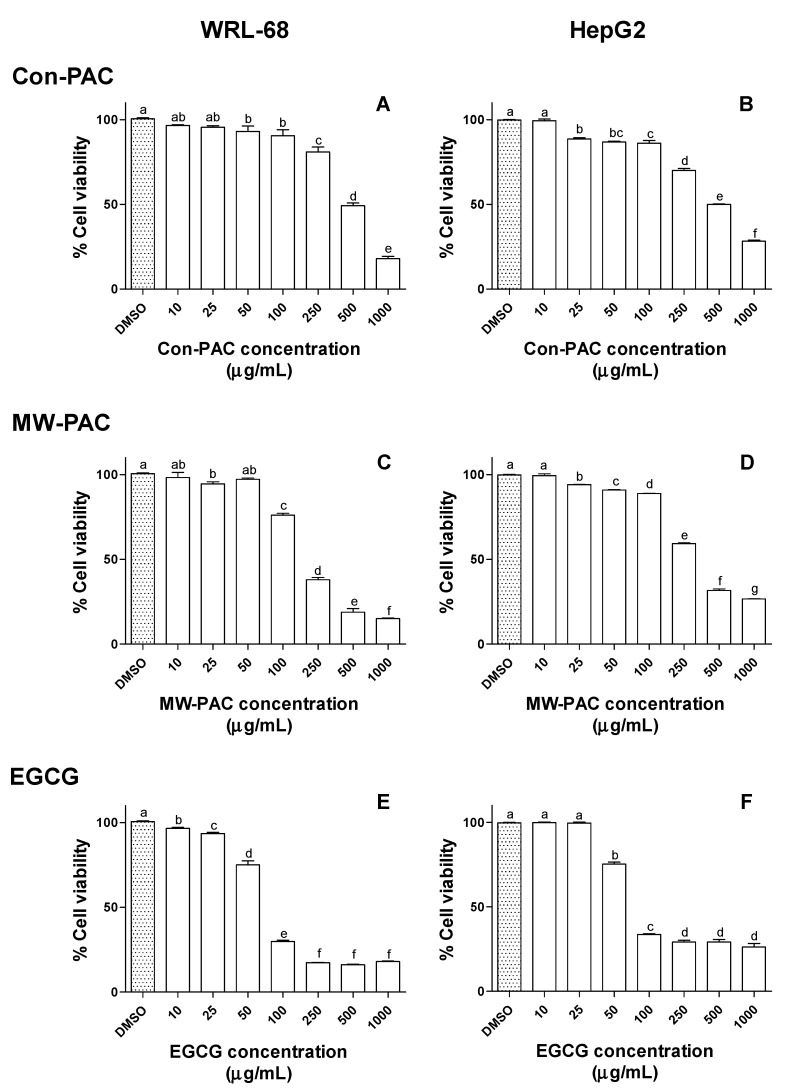
Cell viability (%) of WRL-68 and HepG2 cells exposed to different concentrations of Con-PAC, MW-PAC, and EGCG measured by MTS cell viability assay. WRL-68 and HepG2 cells were exposed to 10–1000 µg/mL concentrations of Con-PAC (**A** and **B**, respectively), MW-PAC (**C** and **D**, respectively), and EGCG (**E** and **F**, respectively) for 24 h. Cell viability of the WRL-68 and HepG2 cells was measured by incubating (3 h at 37 °C) the cells with MTS cell viability reagent. Results presented are means ± SD of three independent experiments. Means sharing the same letter are not significantly different at the 5% level. Con-PAC: PACs extracted by conventional method; MW-PAC: PACs extracted by microwave-assisted method; EGCG, epigallocatechin gallate.

**Figure 6:**
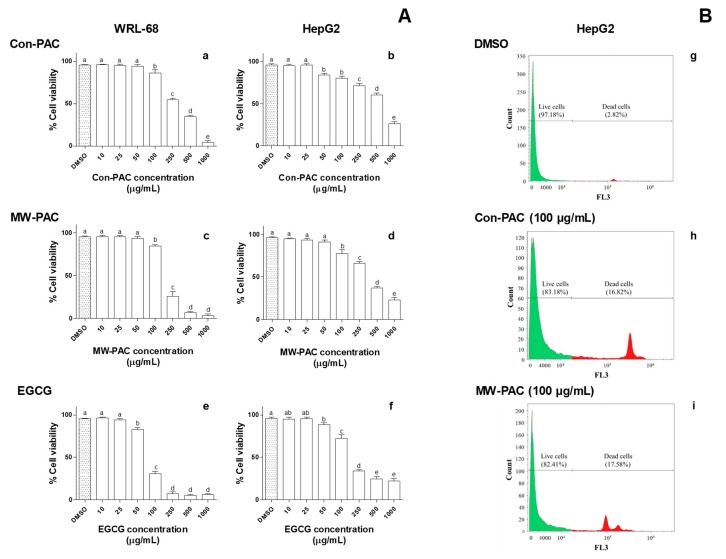
Cell viability (%) of WRL-68 and HepG2 cells exposed to different concentrations of Con-PAC, MW-PAC, and EGCG measured by 7-AAD-stained flow cytometry (**A**) and FL3 histograms of the HepG2 cells (**B**) treated with 100 µg/mL of PAC. WRL-68 and HepG2 cells were exposed to 10–1000 µg/mL concentrations of Con-PAC, MW-PAC, and EGCG for 24 h. Cells were stained with 7-AAD stain in the dark (5 min) and analyzed by flow cytometry under the FL-3 filter. FACS data were processed by Kaluza Analysis (version 2.1) flow cytometry analysis software. Results presented are means ± SD of three independent experiments. Means sharing the same letter are not significantly different at the 5% level. PACs: proanthocyanidins; Con-PAC: proanthocyanidins extracted by the conventional method; MW-PAC: proanthocyanidins extracted by microwave-assisted method; EGCG, epigallocatechin gallate.

The potential anticancer activity of MW-PAC and Con-PAC was measured by the means of capacity to kill hepatocarcinoma (HepG2) cells in comparison to normal (non-malignant) hepatic (WRL-68) cells. The Con-PAC-, MW-PAC-, and EGCG-mediated cytotoxicity were concentration-dependent for both cell lines (Figure 5 and Figure 6). Con-PAC exhibited significant toxicity in both cell lines at concentrations of 250 µg/mL and higher. Cytotoxicity of MW-PAC in both cell lines was similar to that of the Con-PAC, causing higher toxicity at the 250 µg/mL concentration level. The EGCG-mediated cytotoxicity was intense compared to the Con-PAC and MW-PAC. A drastic reduction in % cell viability was observed for EGCG in both cell lines at the concentration of 100 µg/mL. A beneficial effect of selective cytotoxicity in hepatocarcinoma cells (HepG2) compared to healthy hepatic cells (WRL 68) was not observed for either Con-PAC or MW-PAC. 

## 4. Discussion 

Wine production residue has been the subject of extensive research due to its high content of bioactives with important biological functions. Among the different wine industry byproducts, grape seeds contain the highest amount of total phenolic compounds. Catechins and their isomers and polymers are the main phenolic components in grape seeds [27]. Due to their natural antioxidant abilities, GSE can be employed as a functional ingredient in value-added food and nutraceutical products. However, the biological activities of PACs from grape seeds are often confounded by their seemingly low bioavailability. In the present study, MAE was successfully used to extract PACs from grape seeds with enhanced monomeric catechins (increased from 2.10 ± 0.13 mg/g DW to 8.15 ± 0.20 mg/g DW). Advanced graphical and numerical optimizations were run to determine the optimum levels of studied extraction conditions with desirable levels of monomeric catechins and PAC. Under the optimum settings (170 °C; 94% ethanol; 55 min) for maximizing total monomeric catechins, replacing conventional extraction by MAE led to a higher yield of PACs (5.8-fold greater than Con) and monomeric catechins (3.9-fold greater than Con). The higher yields of monomeric catechins obtained for MW-PAC were likely due to extensive PAC depolymerization under the MAE conditions. Higher yields of PACs have also been observed by others [17,18] when combining an MW pretreatment with two-phase aqueous extraction of polyphenols from grape seeds. However, the experimental value for total monomeric catechins (8.15 ± 0.20 mg/g) was lower than the predicted value (18.3 mg/g DW), and the experimental value for total PAC (56.37 ± 8.37 mg CE/g DW) was higher than the predicted value (43.7 mg CE/g DW). Recently, the monograph of green tea was included in the European Pharmacopoeia, wherein green tea is standardized for caffeine content (min 1.5%) and for the total content of catechins, expressed as EGCG (min 8%) [28]. The total monomeric catechin content of the extract by MW in this study was over this standard. As far as we are aware, this is the first study to report microwave irradiation to increase the yields of monomeric catechins through depolymerization of the PACs of grape seeds.

Previous studies have suggested that the content of flavanols from the diet could significantly contribute to their total antioxidant capacities [29]. In the current study, the antioxidant potential of the GSE obtained by MAE and conventional extraction methods were compared. MW-PAC showed higher antioxidant capacity than that of Con-PAC. The correlation analysis showed a significant linear correlation between PAC content and FRAP, indicating that the flavanols in GSE were substantially responsible for the antioxidant capacity.

Dietary flavanols, in addition to their antioxidant effects, have been reported to exert anti-hyperglycemic effects by binding to glucose transporters and competitive inhibition of digestive enzymes [30]. α-Glucosidase is one of the important carbohydrate-hydrolyzing enzymes that digest dietary starch and degrade oligosaccharides to glucose, resulting in postprandial glucose surge. Therefore, inhibition of α-glucosidase activities is one of the primary approaches used to manage hyperglycemic conditions of type 2 diabetic patients. The possibility of clinical use of such inhibitors for diabetic or obese patients has been suggested using acarbose, which has been shown to effectively reduce the intestinal absorption of sugars in humans. MW-PAC exhibited greater α-glucosidase inhibition (IC_50_ of 259.0 ± 30.8 mg/L) than Con-PAC (IC_50_ of 399.1 ± 48.9 mg/L), suggesting that depolymerization of PACs to monomeric catechins enhanced their biological activity. This observation also agrees with the reports that polyphenols are effective inhibitors of α-glucosidase [31]. Interestingly, the activities of both MW-PAC and Con-PAC were comparable to the drug acarbose’s IC_50_ value (347.4 ± 43.5 mg/L). 

The cytotoxicity of Con-PAC and MW-PAC in HepG2 and WRL-68 cells was evaluated. Both Con-PAC and MW-PAC showed similar cytotoxicity in HepG2 and WRL-68 cells. Con-PAC and MW-PAC did not cause relatively higher toxicity in HepG2 cancer cells over WRL-68 normal cells. However, further studies can be recommended to compare the biological activities of MW-PAC at different degrees of depolymerization to prevent or reduce cancer initiation and progression. The high cytotoxicity of EGCG compared to the extracted PACs could have been due to the formation of H_2_O_2_ in the cell culture media [32]. 

## 5. Conclusions

Overall, this is the first report of an application of microwaves as the energy source for achieving PAC depolymerization. The present results showed that MAE is more effective than the conventional method for recovering monomeric catechins and PACs from grape seed powder. PACs are partially depolymerized under the conditions of high ethanol concentration (94%), high temperature (170 °C), and 55 min extraction. The product of MAE showed higher antioxidant capacity and α-glucosidase-inhibitory activities than that of the conventional method, due to the increase of monomeric catechins. The present findings suggest the potential use of industrial byproducts from grape-processing industries as a renewable resource of functional food ingredients and nutraceuticals. 

## Figures and Tables

**Table 1:** Central composite design (CCD) arrangement for microwave-assisted extraction of PACs from grape seed.

Run Order	Temperature (°C)	Time (min)	Ethanol Concentration (%)	Total Monomeric Catechins (mg/g DW)	Total PACs (mg CE/g DW)
1	110 (−1.68)	30 (0)	60 (0)	11.18	108.22
2	120 (−1)	15 (−1)	40 (−1)	8.11	72.12
3	120 (−1)	15 (−1)	80 (+1)	11.72	99.92
4	120 (−1)	45 (+1)	40 (−1)	6.75	83.98
5	120 (−1)	45 (+1)	80 (+1)	11.65	104.18
6	140 (0)	30 (0)	60 (0)	11.03	106.87
7	140 (0)	30 (0)	60 (0)	9.31	107.03
8	140 (0)	30 (0)	60 (0)	9.71	106.83
9	140 (0)	30 (0)	60 (0)	9.26	107.81
10	140 (0)	30 (0)	60 (0)	8.83	103.46
11	140 (0)	30 (0)	60 (0)	9.05	107.58
12	140 (0)	30 (0)	26.4 (−1.68)	3.07	24.44
13	140 (0)	30 (0)	93.6 (+1.68)	10.64	85.48
14	140 (0)	5 (−1.68)	60 (0)	10.58	97.90
15	140 (0)	55 (+1.68)	60 (0)	9.85	90.28
16	160 (+1)	15 (−1)	40 (−1)	8.52	66.58
17	160 (+1)	15 (−1)	80 (+1)	13.84	104.64
18	160 (+1)	45 (+1)	40 (−1)	7.00	38.27
19	160 (+1)	45 (+1)	80 (+1)	15.05	90.81
20	170 (+1.68)	30 (0)	60 (0)	10.41	66.39

The CCD-coded values of temperature, time, and ethanol concentration are shown in brackets; run orders 6 to 11 represent the experiment at the center points repeated six times to allow the estimation of the error variance needed for ANOVA; total monomeric catechins included catechin, epicatechin, and EGC; PACs: proanthocyanidins; CE: catechin equivalents.

**Table 2:** Analysis of variance (ANOVA) *p*-values for the second-order response surface model for total monomeric catechins and total PAC content.

Source of Variation	Total Monomeric Catechins	Total PACs
Temp	0.179	0.001
Time	0.439	0.099
EthConc	0.001	0.001
Temp × Temp	0.020	0.005
Time × Time	0.126	0.075
EthConc × EthConc	0.015	0.001
Temp × Time	0.698	0.005
Temp × EthConc	0.112	0.026
Time × EthConc	0.181	0.683
Adjusted R^2^	85.2%	94.2%

Temp × Temp, Time × Time, and EthConc × EthConc represent the quadratic components of the model; total monomeric catechins included catechin, epicatechin, and EGC; PACs: proanthocyanidins.

**Table 3:** Optimum settings of temperature, ethanol concentration, and time that maximized both total monomeric catechins (mg/g DW) and total PACs (mg CE/g DW). The optimum (maximum) values were obtained by maximizing each response variable separately.

Factor	Optimum Conditions for Total Monomeric Catechins	Optimum Conditions for Total PACs
Temperature	170 °C	120.3 °C
Ethanol concentration	94%	67.9%
Time	55 min	41.4 min
Predicted concentration under the optimum condition for each analyte	18.3 mg/g DW	113.6 mg CE/g DW
Predicted concentration of total monomeric catechins under the optimum condition of total PACs	10.7 mg/g DW	n/a
Predicted concentration of total PACs under the optimum condition of total monomeric catechins	n/a	43.7 mg CE/g DW

Total monomeric catechins include catechin, epicatechin, and EGC; PACs: proanthocyanidins; CE: catechin equivalents; n/a: not applicable; DW, dry weight.

## References

[1] Borges G., Ottaviani J.I., van der Hooft J.J.J., Schroeter H., Crozier A. (2018). Absorption, metabolism, distribution and excretion of (−)-epicatechin: A review of recent findings. Mol. Asp. Med..

[2] Fan F.Y., Sang L.X., Jiang M. (2017). Catechins and their therapeutic benefits to inflammatory bowel disease. Molecules.

[3] Fathima A., Rao J.R. (2016). Selective toxicity of catechin—A natural flavonoid towards bacteria. Appl. Microbiol. Biotechnol..

[4] Anantharaju P.G., Gowda P.C., Vimalambike M.G., Madhunapantula S.V. (2016). An overview on the role of dietary phenolics for the treatment of cancers. Nutr. J..

[5] Chang H.P., Sheen L.Y., Lei Y.P. (2015). The protective role of carotenoids and polyphenols in patients with head and neck cancer. J. Chin. Med. Assoc..

[6] Chen W., Becker T., Qian F., Ring J. (2014). Beer and beer compounds: Physiological effects on skin health. J. Eur. Acad. Dermatol. Venereol..

[7] Losada-Echeberria M., Herranz-Lopez M., Micol V., Barrajon-Catalan E. (2017). Polyphenols as promising drugs against main breast cancer signatures. Antioxidants.

[8] Moga M.A., Dimienescu O.G., Arvatescu C.A., Mironescu A., Dracea L., Ples L. (2016). The role of natural polyphenols in the prevention and treatment of cervical cancer—An overview. Molecules.

[9] Vezza T., Rodriguez-Nogales A., Algieri F., Utrilla M.P., Rodriguez-Cabezas M.E., Galvez J. (2016). Flavonoids in inflammatory bowel disease: A review. Nutrients.

[10] Martin M.A., Goya L., Ramos S. (2013). Potential for preventive effects of cocoa and cocoa polyphenols in cancer. Food Chem. Toxicol..

[11] Zhao Y., Hu X., Zuo X., Wang M. (2018). Chemopreventive effects of some popular phytochemicals on human colon cancer: A review. Food Funct..

[12] Parmar I., Rupasinghe H.P.V., Brar S.K., Kaur S., Dhillon G.S. (2014). Proanthocyanidins in cranberry and grape seeds: Metabolism, bioavailability and biological activity. Nutraceuticals and Functional Foods: Natural Remedy.

[13] Bernatoniene J., Kopustinskiene D.M. (2018). The role of catechins in cellular responses to oxidative stress. Molecules.

[14] Ma Z.F., Zhang H. (2017). Phytochemical constituents, health benefits, and industrial applications of grape seeds: A mini-review. Antioxidants.

[15] Bizzi C.A., Pedrotti M.F., Silva J.S., Barin J.S., Nóbrega J.A., Flores E.M.M. (2017). Microwave-assisted digestion methods: Towards greener approaches for plasma-based analytical techniques. J. Anal. At. Spectrom..

[16] Radojković M., Moreira M.M., Soares C., Fátima Barroso M., Cvetanović A., Švarc-Gajić J., Morais S., Delerue-Matos C. (2018). Microwave-assisted extraction of phenolic compounds from *Morus nigrab* leaves: Optimization and characterization of the antioxidant activity and phenolic composition. J. Chem. Technol. Biotechnol..

[17] Dang Y.-Y., Zhang H., Xiu Z.-L. (2014). Microwave-assisted aqueous two-phase extraction of phenolics from grape (Vitis vinifera) seed. J. Chem. Technol. Biotechnol..

[18] Romero-Diez R., Matos M., Rodrigues L., Bronze M.R., Rodriguez-Rojo S., Cocero M.J., Matias A.A. (2019). Microwave and ultrasound pre-treatments to enhance anthocyanins extraction from different wine lees. Food Chem..

[19] Dambergs R.G., Mercurio M.D., Kassara S., Cozzolino D., Smith P.A. (2012). Rapid measurement of methyl cellulose precipitable tannins using ultraviolet spectroscopy with chemometrics: Application to red wine and inter-laboratory calibration transfer. Appl. Spectrosc..

[20] Benzie I.F.S., Strain J.J. (1999). Ferric reducing/antioxidant power assay: Direct measure of total antioxidant activity of biological fluids and modified version for simultaneous measurement of total antioxidant power and ascorbic acid concentration. Methods Enzymol..

[21] Watanabe J., Kawabata J., Kurihara H., Niki R. (2014). Isolation and identification of α-glucosidase inhibitors from Tochu-cha (*Eucommia ulmoides*). Biosci. Biotechnol. Biochem..

[22] Thilakarathna W.P.D.W., Rupasinghe H.P.V. (2019). Microbial metabolites of proanthocyanidins reduce chemical carcinogen-induced DNA damage in human lung epithelial and fetal hepatic cells in vitro. Food Chem. Toxicol..

[23] Myers R.H., Montgomery D.C., Anderson-Cook C.M. (2016). Response Surface Methodology: Process and Product Optimization Using Designed Experiments.

[24] Montgomery D.C. (2017). Design and Analysis of Experiments.

[25] Majewska M., Lewandowska U. (2017). The chemopreventive and anticancer potential against colorectal cancer of polyphenol-rich fruit extracts. Food Rev. Int..

[26] Ramos S., Martin M.A., Goya L. (2017). Effects of cocoa antioxidants in type 2 diabetes mellitus. Antioxidants.

[27] Yammine S., Brianceau S., Manteau S., Turk M., Ghidossi R., Vorobiev E., Mietton-Peuchot M. (2018). Extraction and purification of high added value compounds from by-products of the winemaking chain using alternative/nonconventional processes/technologies. Crit. Rev. Food Sci. Nutr..

[28] Council of Europe (2018). Green tea (*Camelliae sinensis* non fermentata folia). European Pharmacopoeia.

[29] Valls J., Agnolet S., Haas F., Struffi I., Ciesa F., Robatscher P., Oberhuber M. (2017). Valorization of Lagrein grape pomace as a source of phenolic compounds: Analysis of the contents of anthocyanins, flavanols and antioxidant activity. Eur. Food Res. Technol..

[30] Sekhon-Loodu S., Rupasinghe H.P.V. (2019). Evaluation of antioxidant, antidiabetic and antiobesity potential of selected traditional medicinal plants. Front. Nutr..

[31] Wang H., Liu T., Huang D. (2013). Starch hydrolase inhibitors from edible plants. Adv. Food Nutr. Res..

[32] Weisburg J.H., Weissman D.B., Sedaghat T., Babich H. (2004). In vitro cytotoxicity of epigallocatechin gallate and tea extracts to cancerous and normal cells from the human oral cavity. Basic Clin. Pharmacol. Toxicol..

